# Exposure to common infections may shape basal immunity and potentially HIV-1 acquisition amongst a high-risk population in Coastal Kenya

**DOI:** 10.3389/fimmu.2023.1283559

**Published:** 2024-01-11

**Authors:** Lynn Fwambah, Cheryl Andisi, Claire Streatfield, Rachel Bromell, Jonathan Hare, Joakim Esbjörnsson, Thumbi Ndung’u, Eduard J. Sanders, Amin S. Hassan, Eunice Nduati

**Affiliations:** ^1^ Kenya Medical Research Institute (KEMRI)/Wellcome Trust Research Programme, Kilifi, Kenya; ^2^ Department of Biological Sciences, Pwani University, Kilifi, Kenya; ^3^ International AIDS Vaccine Initiative (IAVI) Human Immunology Laboratory, Imperial College, London, United Kingdom; ^4^ International AIDS Vaccine Initiative (IAVI), New York, NY, United States; ^5^ Department of Translational Medicine, Lund University, Lund, Sweden; ^6^ Nuffield Department of Clinical Medicine, University of Oxford, Oxford, United Kingdom; ^7^ Africa Health Research Institute (AHRI), Durban, KwaZulu-Natal, South Africa; ^8^ Human Immunodeficiency Virus (HIV) Pathogenesis Programme, The Doris Duke Medical Research Institute, University of KwaZulu-Natal, Durban, South Africa; ^9^ Ragon Institute of Massachusetts General Hospital, Massachusetts Institute of Technology and Harvard University, Cambridge, MA, United States; ^10^ Division of Infection and Immunity, University College London, London, United Kingdom; ^11^ The Aurum Institute, HIV Division, Johannesburg, South Africa

**Keywords:** HIV acquisition, infections, exposure, cytokines, chemokines

## Abstract

**Introduction:**

The impact of exposure to endemic infections on basal immunity and susceptibility to HIV-1 acquisition remains uncertain. We hypothesized that exposure to infections such as cytomegalovirus (CMV), malaria and sexually transmitted infections (STIs) in high-risk individuals may modulate immunity and subsequently increase susceptibility to HIV-1 acquisition.

**Methods:**

A case-control study nested in an HIV-1 negative high-risk cohort from Coastal Kenya was used. Cases were defined as volunteers who tested HIV-1 positive during follow-up and had a plasma sample collected 3 ± 2 months prior to the estimated date of HIV-1 infection. Controls were individuals who remained HIV-1 negative during the follow-up and were matched 2:1 to cases by sex, age, risk group and follow-up time. STI screening was performed using microscopic and serologic tests. HIV-1 pre-infection plasma samples were used to determined exposure to CMV and malaria using enzyme-linked immunosorbent assays and to quantify forty-one cytokines and soluble factors using multiplexing assays. Multiplexing data were analyzed using principal component analysis. Associations between cytokines and soluble factors with subsequent HIV-1 acquisition were determined using conditional logistic regression models.

**Results and discussion:**

Overall, samples from 47 cases and 94 controls were analyzed. While exposure to malaria (p=0.675) and CMV (p=0.470) were not associated with HIV-1 acquisition, exposure to STIs was (48% [95% CI, 33.3 – 63] vs. 26% [95% CI, 17.3 – 35.9]. Ten analytes were significantly altered in cases compared to controls and were clustered into four principal components: PC1 (VEGF, MIP-1β, VEGF-C and IL-4), PC2 (MCP-1, IL-2 and IL-12p70), PC3 (VEGF-D) and PC4 (Eotaxin-3). PC1, which is suggestive of a Th2-modulatory pathway, was significantly associated with HIV-1 acquisition after controlling for STIs (adjusted odds ratio, (95% CI), p-value: 1.51 [1.14 – 2.00], p=0.004). Elevation of Th2-associated pathways may dampen responses involved in viral immunity, leading to enhanced susceptibility to HIV-1 acquisition. Immunomodulatory interventions aimed at inhibiting activation of Th2-associated pathways may be an additional strategy to STI control for HIV-1 prevention and may reduce dampening of immune responses to vaccination.

## Introduction

Sub-Saharan Africa (sSA) bears the greatest burden of infectious diseases. Persistent pathogens such as helminths, bacteria, parasites and viruses are common (1). The constant exposure to these pathogens may alter one’s immunologic landscape at baseline – henceforth referred as basal immunity (2). It is possible that an altered basal immunity may modulate the acquisition of new infections and impact vaccine responses. For instance, helminth infections are common in sSA and have been reported to induce a state of immune suppression via Th2 and T-regulatory mediated hyporesponsiveness and anergy (3–6). This immunosuppressive profile may dampen the severity of inflammatory conditions such as auto-immune diseases (6), malaria (7), and coronavirus disease 2019 (COVID-19) (4, 8). Contrarily, immunosuppression may dampen immune responses towards other pathogens and vaccines that require a Th1 response (4), and thereby potentially increase the infectivity or severity of infections. *Plasmodium falciparum* (*P. falciparum*), the causative agent for malaria, is another distinct and most common malaria pathogen in sSA. Frequent exposures to *P. falciparum*, may favour a shift of immune responses to a state of tolerance, protecting the host from malaria-induced pathology by down-regulating hyperinflammatory responses (9, 10). Moreover, repeated malaria exposures are associated with a modified immune system in children that is marked by upregulation of interferon-inducible genes, higher levels of IL-10 and enhanced cellular activation (11) and have also been associated with immunosuppression and immune cell exhaustion (12, 13). Cytomegalovirus (CMV) is also a common pathogen both globally and in sSA (14). It typically causes acute disease in naïve and immunocompromised individuals but can also have profound impact on the host immune system. Its immunological imprinting is not only restricted to CMV-specific responses but can also affect immunity against other viral and non-viral infectious agents as well as immunopathological responses (15). Further and in the context of HIV-1 negative high-risk individuals, sexually transmitted infections (STIs) like Chlamydia, Gonorrhoeae and Trichomoniasis are not uncommon (16). Frequent exposures to STIs create mechanical mucosal damage by genital inflammation and ulceration (17, 18) and may also cause an imprint on the immune system (19, 20) that contributes to shaping consecutive immune responses to secondary pathogen exposures. Understanding how exposure to infectious pathogens alters basal immunity and helps shape responses to a secondary infection may inform observed differences in disease burden and vaccine efficacy within-and-between populations. So far, evidence to explain heterogeneity in the distribution of the HIV-1 epidemic remains sparse. It is possible that endemicity of underlying infections within the sSA setting (21) contributes to an altered baseline immune response that predisposes individuals to higher risk of HIV-1 acquisition (21). We hypothesized that exposure to *P. falciparum*, cytomegalovirus (CMV) and STIs results in altered basal immunity which enhances susceptibility to HIV-1 acquisition. We sought to test this hypothesis in a retrospective analysis of data and samples from a HIV-1 high-risk cohort from Coastal Kenya.

## Materials and methods

### Study design

A case-control study nested in a historic HIV-1 high-risk cohort from Coastal Kenya was developed. Briefly, HIV-1 negative high-risk volunteers, either men who have sex with men (MSM), or female sex workers (FSW) aged ≥18 years were recruited and followed up between 2006 and 2011 as part of a multi-country cohort for HIV-1 vaccine preparedness studies (22, 23). During follow-up, volunteers were screened for incident HIV-1 infection using RT-PCR assay, HIV-1 p24 antigen assay and/or HIV-1 specific antibody assays (22). For those who tested positive for HIV-1 infection, an estimated date of infection (EDI) was calculated as follows: 10 days before a positive HIV-1 RNA test (if antibody negative), 14 days before a p24 antigen positive test (if HIV-1 RNA test was missing and antibody negative) and mid-way of a last negative test and first positive HIV-1 specific antibody test (if RNA and p24 antigen tests were missing) (24).

### Study population

For the purpose of our study, cases were defined as volunteers who tested HIV-1 positive during follow-up, while controls were defined as volunteers who remained HIV-1 negative at the end of a similar follow-up period. For cases, plasma samples collected 3 ± 2 months prior to the EDI were retrieved. Controls were matched 2:1 to cases based on age, sex, risk group, follow-up duration in the study and having plasma samples collected at around the same calendar date as that of the index case ±2 months.

### Laboratory methods

#### Measurement of exposure to sexually transmitted infections

Available data on sexually transmitted infections (STIs) were extracted from the cohort database. STI data collected up to six months prior to the EDI (for cases) or a matched follow up period (for controls) were used as measures of exposure to STIs. Overall, six STIs including gonorrhea, chlamydia, hepatitis B, syphilis, trichomoniasis, and yeast infection, were diagnosed using a mix of clinical and laboratory methods as previously described (25). In brief, chlamydia and gonorrhea infections were diagnosed using syndromic screening for urethral/rectal discharge and confirmed using Gram stain. Trichomoniasis and yeast infections (Candidiasis) were diagnosed using syndromic screening for vaginal/rectal discharge, dysuria and itchiness, and confirmed using wet prep microscopy. Hepatitis B was screened clinically based on yellowing of the skin, eyes and dark urine, and confirmed with serology tests for hepatitis B virus antigens and antibodies. Syphilis was diagnosed using syndromic screening of genital/rectal sores and confirmed using Rapid Plasma Reagin (RPR) and *Treponema pallidum* Hemagglutinin (TPHA) tests. A composite STI variable, defined as volunteers who tested positive for any of the six STIs during the defined window period, was generated and carried forward to downstream analyses.

#### Measurement of exposure to malaria parasite (P. falciparum)

Plasma samples were analyzed for the presence of IgG antibodies against malaria schizonts as a measure of cumulative exposure to malaria, as previously reported (26). Therefore, exposure is defined here as the serological detection of pathogen-specific antibodies. Schizont extract was prepared from the NF54 malaria parasite strain. ELISA was conducted by coating each well with schizont extract lysate. Dilution series of pooled hyper-immune serum that had previously been determined, were added to each plate for the generation of a standard curve. Malaria seropositivity was determined based on a cut-off of negative control plus two standard deviations (SDs). In addition, arbitrary units (AU) were assigned to the standard dilution curve from which AU concentrations of the samples tested were interpolated.

#### Measurement of exposure to cytomegalovirus

Plasma IgG antibodies against CMV were determined as a measure of previous exposure to the virus. Commercial ELISA plates (EUROIMMUN Medizinische, Germany) pre-coated with lysed MRC-5 cells infected with the CMV AD169 strain were used. In brief, 100 µl of calibrators, controls and diluted samples were added to the plate and processed as per the manufacturer’s instructions (27). The optical density readings from the samples were read at 450 nm on a Synergy 4 (Bio Tek) plate reader. Anti-CMV antibody concentrations in each sample were quantified from a standard curve constructed using the calibrators.

#### Measurement of basal cytokines and other soluble factors

Measurement of cytokines and soluble factors is considered reflective of the overall basal immune microenvironment. Multiplexing was done using the Meso Scale Discovery (MSD) human V-Plex multi-spot assay kits, which is designed to reduce sample handling and allow for simultaneous processing of multiple samples and analytes, hence reduce variability (28). In brief, 25 µl of prepared calibrators and samples were added to the wells in duplicates and the plates were processed according to manufacturer’s instructions (28). After processing, the plates were read on an MSD Quickplex SQ120 imager, and concentrations interpolated from a standard curve. The respective upper (ULOQ) and lower (LLOQ) limits of quantification for each analyte were also inferred. Analyte concentrations below the LLOQ were assigned half the LLOQ concentration, whilst those above the ULOQ were assigned the ULOQ value, as previously reported (29). Duplicate observations were aggregated into a mean value and used in downstream analysis.

### Data analysis

Socio-demographic characteristics of cases and controls were compared using the Wilcoxon rank-sum and chi-square tests for continuous and categorical variables, respectively. Malaria and CMV antibody titres were compared between cases and controls using Wilcoxon rank-sum tests. Correlations between malaria and CMV antibody titres with cytokines and soluble factors were assessed using Pearson correlation tests. The composite STI variable was compared between cases and controls using the chi-square test and associations with cytokines and soluble factors assessed using the Wilcoxon rank-sum tests.

Comparison of basal cytokines and other soluble factors between cases and controls were initially assessed using Wilcoxon rank-sum tests. From an exploratory model-building approach, cytokines and other soluble factors with a p-value<0.1 between cases and controls were carried forward to principal component analysis (PCA) to elucidate clustering patterns that may better explain underlying common immune-modulatory pathways. Subsequently, univariable and multivariable conditional logistic regression models were used to assess associations between the different principal components and HIV-1 acquisition. Associations with a p-value <0.05 were considered statistically significant.

Statistical analyses were done using Stata/IC version 17 (StataCorp LP, California). Graphical representations were generated using GraphPad Prism version 7.0 (GraphPad Software, California). A 2-sided type I error of 10% was considered acceptable to inform model building, 5% acceptable for statistical significance, and no formal corrections were made for multiple comparisons, as is consistent with previous publications (29–31).

### Ethical approval and volunteer consent

The study received ethical approval from the Kenya Medical Research Institute (KEMRI) Science and Ethics Review Unit (SERU protocol no. 894). Volunteers provided written informed consent for the sharing and use of data and samples for research. Volunteers who acquired HIV-1 infection during follow-up despite receiving HIV-1 risk-reduction counselling and testing (24), were referred for follow-up HIV-1 care and treatment. STIs were treated according to the Kenyan national treatment guidelines (25).

## Results

### Selection and distribution of volunteers

**Figure 1:**
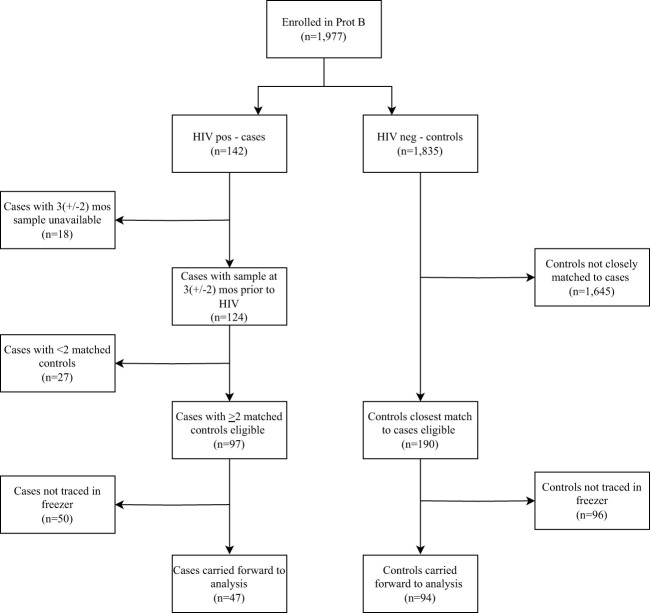
Flow chart of volunteers enrolled into the parent cohort, followed up over time and included in the analysis based on the eligibility criteria. Prot B, Protocol B; HIV +ve, HIV-1 positive; HIV –ve, HIV-1 negative.

Of the 1,977 volunteers enrolled in the parent cohort, 142 tested HIV-1 positive during follow-up. Of these, 47 and 94 met our eligibility criteria for cases and controls, respectively (**Figure 1**). No significant difference in socio-demographic and clinical characteristics were found between cases and controls (**Table 1**).

**Table 1:** Distribution of socio-demographic and clinical characteristics of volunteers at high risk of HIV-1 acquisition from Coastal Kenya (n=141).

Characteristic	Grouping	Cases (n=47)	Controls (n=94)	P-value
**Gender**	Male Female	37 (78.7) 10 (21.3)	74 (78.7) 20 (21.3)	0.999
**Age group in years**	18.0 – 24.9 25.0+	28 (59.6) 19 (40.4)	56 (59.6) 38 (40.4)	0.999
**Risk group**	MSM-W MSM-E MSW-E WSM-E	31 (66.0) 3 (6.4) 3 (6.4) 10 (21.3)	62 (66.0) 6 (6.4) 6 (6.4) 20 (21.3)	0.827
Follow-up time in months (group)*	0 – 5.9 6.0+	27 (57.5) 20 (42.6)	54 (57.5) 40 (42.6)	0.886
**Marital status**	Single Married Widowed	39 (83.0) 4 (8.5) 4 (8.5)	79 (84.0) 6 (6.4) 9 (9.6)	0.425
**Education level**	None Primary Secondary Higher	3 (6.4) 17 (36.2) 23 (48.9) 4 (8.5)	3 (3.2) 47 (50.0) 37 (39.4) 7 (7.5)	0.741
Circumcision status**	No Yes Missing	4 (10.8) 32 (86.5) 1 (2.7)	6 (8.1) 64 (86.5) 4 (5.4)	0.741

MSM-W, men who have sex with men and women; MSM-E, men who have sex with men exclusively; MSW-E, men who have sex with women exclusively/heterosexual male; WSM-E, women who have sex with men exclusively/heterosexual female; IQR, Interquartile range.

*From enrolment into protocol B (parent cohort) to date of sampling.

**Applies for male volunteers only (n=111).

Of the 41 analytes assayed, four (CRP, ICAM-1, VCAM-1 and SAA) had concentrations that were higher than the ULOQ in 95.0%, 97.9%, 97.2%, and 87.9% of volunteers respectively and were therefore excluded from further analysis. Of the remaining 37 analytes, excellent duplicity (between-duplicates correlation coefficient, r>0.9) was observed, except for three analytes (VEGF-C [r=0.72], IL-1β [r=0.38] and IL-13 (r=0.88]) (**Supplementary Figure 1**, **Supplementary Table 1**). The observed sub-par correlation for the three analytes was driven by six outlier datapoints, which were excluded from the analysis. Following the removal of outliers, the between duplicate correlation coefficient for the three analytes was improved (VEGF-C [r=0.98], IL-1β [r=0.93] and IL-13 (r=0.95]) and the analytes carried forward to downstream analyses (**Supplementary Table 1**).

### Magnitude of exposures

#### Exposure to malaria

**Figure 2:**
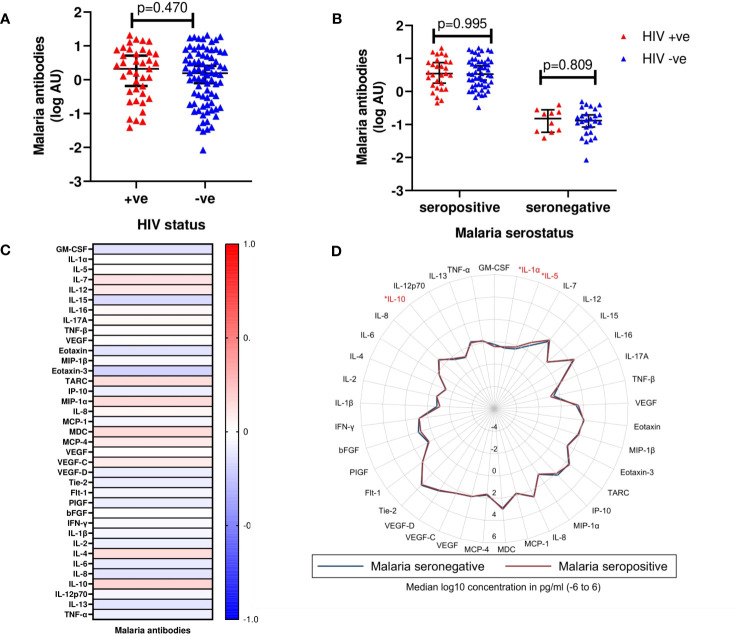
**(A)** Median log_10_ concentrations of malaria antibody titres in cases and controls. Red triangles (HIV +ve) denote volunteers who contracted HIV-1 during follow up (cases). Blue triangles (HIV -ve) represent volunteers who remained HIV-1 negative (controls). Horizontal bars denote median and interquartile range (n=117). *AU, Arbitrary units* **(B)** Median log_10_ concentrations of malaria antibody titres between cases and controls based on malaria serostatus. Red triangles (HIV +ve) and blue triangles (HIV -ve) (n=117). **(C)** Heat map correlating detectable malaria antibody titres and cytokine concentrations. Intensity ranging from red to blue, representing a strong positive correlation (r=+1.0) to a strong negative correlation (r=-1.0). None of the analytes were observed to be significantly different between the two groups. **(D)** Radar plot illustrating differences in cytokines assayed between malaria seronegative and seropositive volunteers. Blue line represents malaria seronegative samples; Red line represents malaria seropositive samples. Cytokines observed to exhibit significant differences are highlighted in red (n=117); p<0.05 [*].

Plasma samples from 117 volunteers (cases (n=39) and controls (n=78) were available for analysis of malaria exposure. Of these, 81 (69.2%) were positive for schizont-specific IgG antibodies. There was no statistically significant difference in the distribution of schizont-specific IgG antibodies between cases and controls (median log_10_ malaria antibodies [IQR]; 0.32 [-0.18 - 0.71] vs 0.19 [-0.02 - 0.32], p=0.470) (**Figure 2A**). When stratified by malaria sero-status, there was also no statistically significant difference in schizont-specific IgG antibodies between cases and controls for neither the malaria sero-positive group (p=0.995) nor the malaria sero-negative group (p=0.809) (**Figure 2B**).

There were no statistically significant correlations between schizont-specific IgG antibodies and any of the 37 analytes included in the analysis (**Figure 2C**, **Supplementary Table 2**). When stratified by malaria sero-status, IL-1α (p=0.034), IL-5 (p=0.023) and IL-10 (p=0.024) were significantly higher in malaria sero-positive than sero-negative volunteers (**Figure 2D**, **Supplementary Table 3**).

#### Exposure to cytomegalovirus

**Figure 3:**
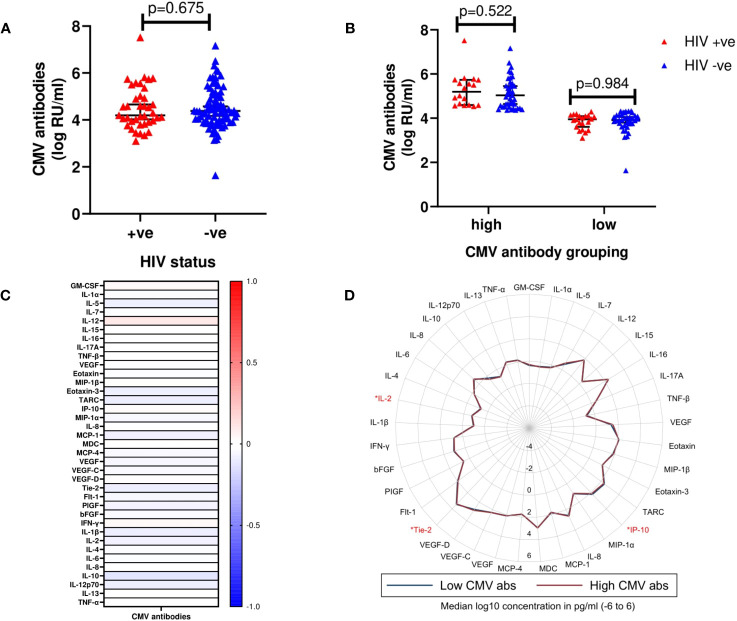
**(A)** Median log_10_ concentrations of CMV antibody titres in cases and controls. Red triangles (HIV +ve) represent volunteers who contracted HIV-1 during follow up (cases). Blue triangles (HIV -ve) represent volunteers who remained HIV-1 negative (controls). Horizontal bars denote median and interquartile range (n=117). *RU/ml, Relative units/ml*. **(B)** Median log_10_ concentrations of high (above median CMV antibody titre) and low (below median CMV antibody titre) CMV antibody titres in cases and controls. **(C)** Heat map displaying correlation between detectable CMV antibody titres and cytokines. Intensity ranging from red to blue, representing a strong positive correlation (r=+1.0) to a strong negative correlation (r=-1.0). None of the analytes were observed to be significantly different between the two groups. **(D)** Radar plot demonstrating differences in cytokines assayed between high (red line) and low (blue line) CMV antibody titres. Cytokines observed to exhibit significant differences are highlighted in red (n=117); p<0.05 [*].

Plasma samples from 115 volunteers (cases (n=39) and controls (n=78) were available for analysis of CMV exposure. Of these, 98.3% had CMV antibody titres above the detection threshold. There was no statistically significant difference in the distribution of CMV antibody titres between cases and controls (median log_10_ CMV antibodies [IQR]; 4.20 [3.48 – 4.54] vs 4.38 [4.10 – 4.64], p=0.675) (**Figure 3A**). Volunteers were then stratified as having either “high” (above median) or “low” (below median) anti-CMV antibody levels. Similarly, no statistically significant differences in CMV antibody titres between cases and controls for neither high (p=0.522) nor low (p=0.984) CMV antibody titre groups were observed (**Figure 3B**).

There were also no significant correlations between CMV antibody titres and any of the 37 analytes included in the analysis (**Figure 3C**, **Supplementary Table 2**). However, using the “high” or “low” stratification, anti-CMV antibody levels, IP-10 (p=0.017), Tie-2 (p=0.017), and IL-2 (p=0.034) were significantly higher in volunteers within the “high” compared to the “low” anti-CMV antibodies group (**Figure 3D**, **Supplementary Table 3**).

#### Exposure to sexually transmitted infections

Overall, 48/141 (34.0%) volunteers were positive for either one or more of the six STIs screened, with gonorrhea being the most common (**Supplementary Figure 2**). Further, 17 (12.1%) volunteers had more than one STI, with a gonorrhea/yeast infection co-infection being most common (**Supplementary Table 4**).

**Figure 4:**
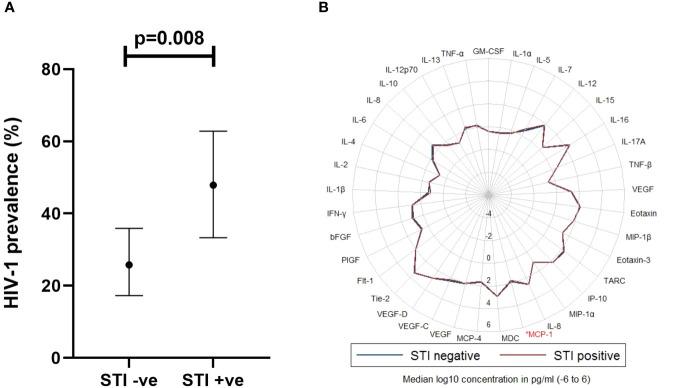
**(A)** Graph illustrating association between STI exposure and HIV-1 acquisition (Chi-square test, p<0.05 considered significant, n=141), where STI +ve or STI -ve was defined as the presence or lack of any STI, respectively in the last 6 months prior to HIV-1 incidence in cases. Horizontal bars denote mean and 95% Confidence Interval. **(B)** Radar plot demonstrating differences in cytokines assayed between STI +ve (red line) and STI -ve (blue line) volunteers. Cytokine observed to exhibit a significant difference is highlighted in red*. STI, sexually transmitted infection; MCP-1, Monocyte chemoattractant protein 1*. Wilcoxon rank sum test, p<0.05 considered significant, (n=141); p<0.05[*].

Volunteers with a history of an STI exposure were more likely to have acquired HIV-1 infection compared to those without (48% [95% CI, 33.3 – 62.8] vs. 26% [95% CI, 17.3 – 35.9] respectively, p=0.008) (**Figure 4A**). There was no statistically significant difference in the prevalence of HIV-1 infection among volunteers with more than one STI, compared to those with one STI, but both had a significantly higher prevalence of HIV-1 infection compared to those without any STI (**Supplementary Figure 4**). Of the 37 analytes, MCP-1 (p=0.036) was significantly elevated in volunteers with a history of an STI exposure, compared to those without (**Figure 4B**).

Overall, all volunteers had a history of exposure to either malaria, CMV or STIs, while 21 (17.9%) had all the three co-infections (**Supplementary Table 5**).

### Association between cytokines and soluble factors with HIV-1 acquisition

**Figure 5:**
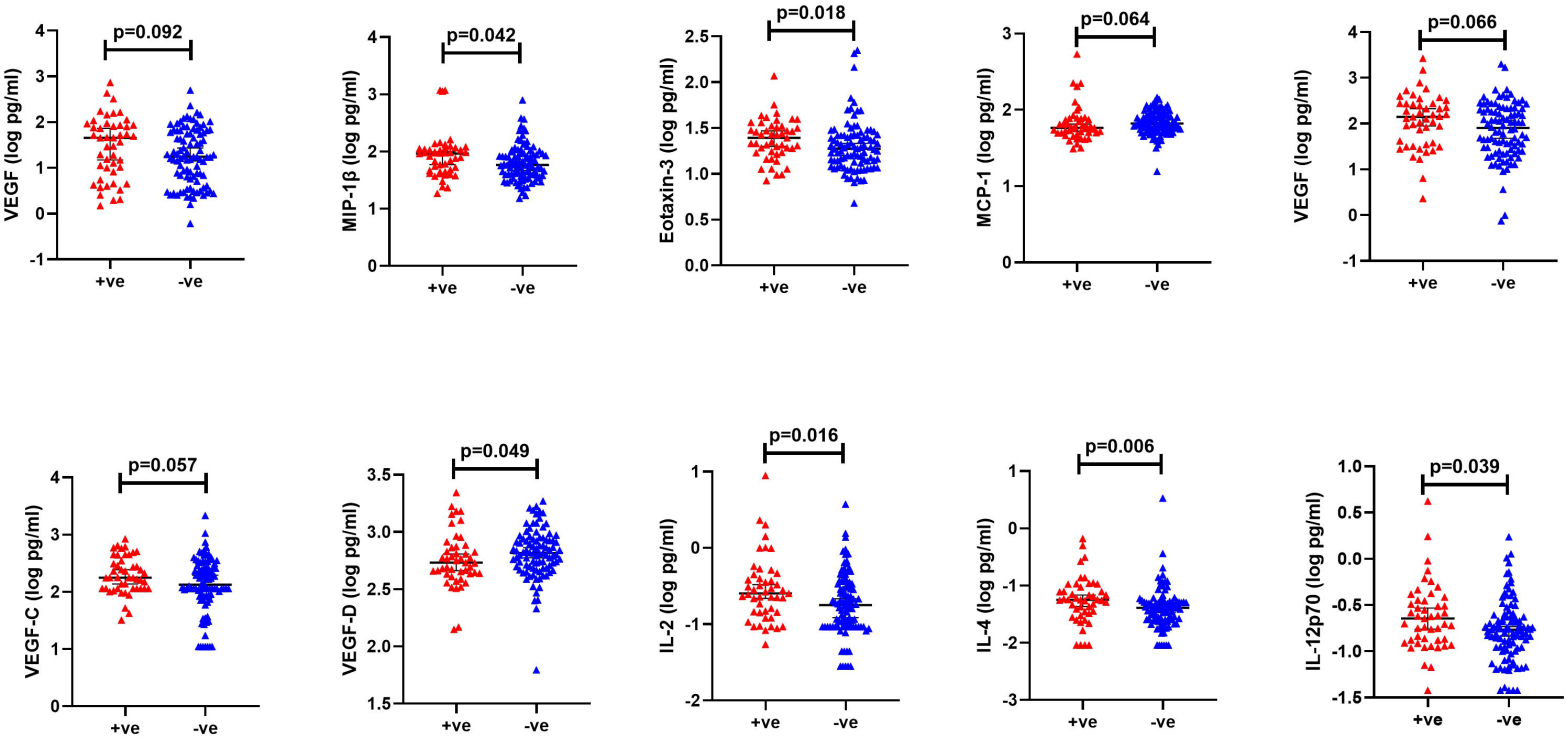
Significantly different cytokine levels between HIV +ve (red): volunteers who contracted HIV-1 during follow up (cases) and HIV -ve (blue): volunteers who remained HIV-1 negative (controls). Horizontal bars denote median and interquartile range.

From an exploratory analysis, VEGF (cytokine panel, p=0.092), MIP-1β (p=0.042), Eotaxin-3 (p=0.018), VEGF (angiogenesis panel, p=0.066), VEGF-C (p=0.057), IL-2 (p=0.016), IL-4 (p=0.006) and IL-12p70 (p=0.039) were elevated in cases, while VEGF-D (p=0.049) and MCP-1 (p=0.064) were higher in controls (**Figure 5**, **Supplementary Figure 3**). After controlling for STIs in a conditional logistic regression model, none of the analytes demonstrated a statistically significant association with HIV-1 acquisition (**Table 2**).

**Table 2:** Conditional logistic regression analysis showing association between cytokines and HIV-1 acquisition (n=141).

Cytokine	Crude Odds Ratio (95% CI)	P-value	*Adjusted Odds Ratio (95% CI)	P-value
**VEGF**	2.94 (1.20 - 7.20)	**0.018**	2.07 (0.72 - 5.91)	0.175
**MIP-1β**	2.88 (0.95 - 8.75)	0.062	…	…
**Eotaxin-3**	3.47 (0.81 - 14.83)	0.093	…	…
**MCP-1**	0.59 (0.06 - 5.67)	0.651	…	…
**VEGF**	1.98 (0.99 - 3.94)	0.054	…	…
**VEGF-C**	3.91 (1.31 - 11.67)	**0.015**	1.79 (0.52 - 6.09)	0.354
**VEGF-D**	0.24 (0.04 - 1.57)	0.137	…	…
**IL-2**	3.11 (1.28 - 7.60)	**0.013**	1.88 (0.57 - 6.18)	0.301
**IL-4**	4.00 (1.24 - 12.90)	**0.020**	1.09 (0.19 - 6.17)	0.922
**IL-12p70**	4.69 (1.31 - 16.78)	**0.017**	1.54 (0.22 - 10.97)	0.666

*Adjusted only for sexually transmitted infections (STIs) as exposure to malaria and CMV showed no association with HIV-1 acquisition.

Bold values denote significant p-values.

**Figure 6:**
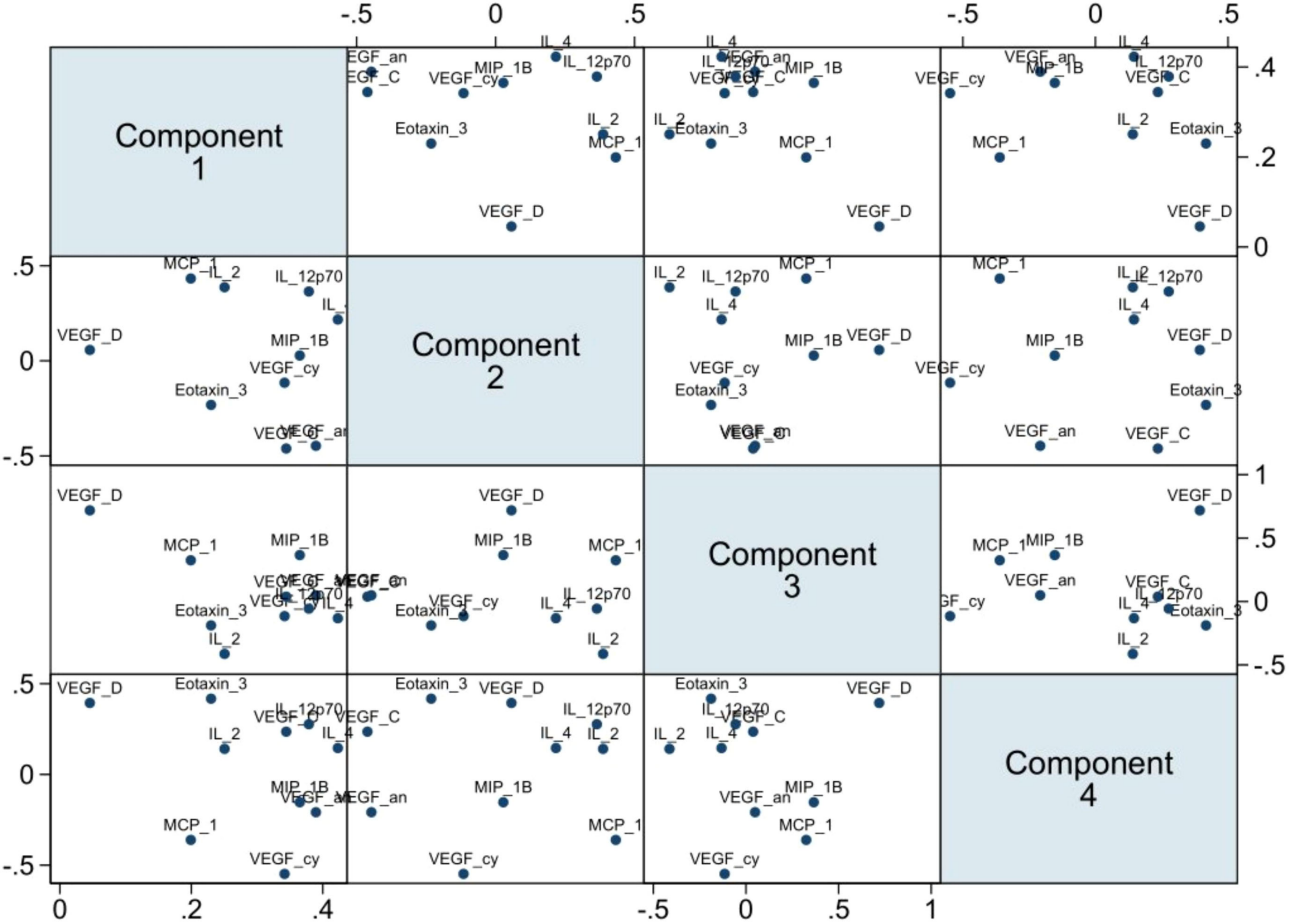
PCA multi-plot showing clustering and relatedness of the ten analytes. Bottom axis from the left: PC1, PC2, PC3 scores. Left axis from the top: PC2, PC3, PC4 scores. Top axis from the left: PC2, PC3, PC4 scores. Right axis from the top: PC1, PC2, PC3 scores (n=141).

The ten analytes were carried forward into a principal component analysis (PCA) after assessing for sufficiency in their variation to justify the approach (Kaiser-Meyer-Olkin, KMO=0.69). A four-component analysis was deemed appropriate (Eigenvalues>1). Overall, the ten analytes clustered into the four components as follows: PC1 (VEGF, MIP-1β, VEGF-C and IL-4), PC2 (MCP-1, IL-2 and IL-12p70), PC3 (VEGF-D) and PC4 (Eotaxin-3) (**Figure 6**). In a conditional logistic regression, PC1 was significantly associated with HIV-1 acquisition after controlling for STIs (adjusted odds ratio, (95% CI), p-value: 1.51 [1.14 – 1.99], p=0.004 (**Table 3**).

**Table 3:** Conditional logistic regression analysis assessing association between the four principal components and HIV-1 acquisition (n=141).

Principal Components	Crude Odds Ratio (95% CI)	P-value	*Adjusted Odds Ratio (95% CI)	P-value
**PC1**	1.54 (1.18 - 2.01)	**0.002**	1.51 (1.14 – 2.00)	**0.004**
**PC2**	0.93 (0.64 - 1.35)	0.695	…	…
**PC3**	0.64 (0.43 - 0.95)	**0.026**	0.74 (0.49 - 1.14)	0.170
**PC4**	1.09 (0.74 – 1.62)	0.652	…	…

*Adjusted only for sexually transmitted infections (STIs) as exposure to malaria and CMV showed no association with HIV-1 acquisition.

Bold values denote significant p-values.

## Discussion

Overall and consistent with literature, our findings suggest that there was high exposure to malaria and CMV in this study population from Coastal Kenya, and that exposure to STIs increased susceptibility to HIV-1 acquisition amongst these HIV-1 high-risk individuals (16, 32). From a basal immunity standpoint, interestingly, PC1 analytes (MIP-1β, VEGF, VEGF-C and IL-4), which are suggestive of a Th2 associated immune profile (33–35), also independently increased susceptibility to HIV-1 acquisition, even after controlling for STIs. VEGF and VEGF-C play a major role in lymphangiogenesis and in tissue repair. They may stimulate the generation of endothelial cells for leukocyte travel to local sites of infection (36), and in turn induce production of chemokines, including MIP-1β (37, 38). MIP-1β can attract and activate monocytes, T-cells and B-cells to sites of inflammation (39), and promotes IL-4 production in T-cells *ex vivo* (33). IL-4 also acts as a key regulator of immune responses and can also promotes the differentiation of naïve T-cells to Th2 cells, favouring production of IgE-secreting B-cells and activation of M2 macrophages (40). M2 macrophages are HIV-1 target cells as they express CD4 and CCR5, and can therefore be infected by the R5 HIV-1 strain (41). As MIP-1β is a chemokine ligand for CCR5, it may act as an endogenous inhibitor of infection with M-tropic-HIV-1 strains and reduce disease progression (42, 43), contrarily prior to HIV-1 acquisition, higher levels of MIP-1β has been associated with increased susceptibility to HIV-1 acquisition (44). In agreement, our data showed that elevated MIP-1β levels were a risk factor for HIV-1 acquisition.

MCP-1 stood out to be significantly elevated in individuals with a history of STI exposure compared to those without. MCP-1 is a chemokine responsible for attracting monocytes, macrophages and natural killer cells to inflammation sites (45). Although macrophages offer first-line defence during bacterial and viral infection and together with NK cells secrete the anti-viral cytokine IFN-γ in response to pathogens (45). This may be key in clearance of hepatitis B and bacterial STIs (45) but may be limited in HIV-1 due to the dual role of macrophages in acute HIV-1 infection where they are susceptible to infection and contribute to the spreading of the virus to other tissues (46).

Malaria and CMV threshold positivity were at 69.2% and 99.2% respectively, which is consistent with previous reports (14, 47) and underscores their endemicity in this population. Exposure to these infections were similar in cases and controls. Interleukin-5, IL-1α, and IL-10 were significantly higher in malaria seropositive than seronegative volunteers. This observation may be indicative of chronic exposure to malaria parasites among seropositive volunteers, as these cytokines are involved in clearance of the malaria parasite (48–50). While IP-10, Tie-2 and IL-2 were significantly higher in those with high, compared to low CMV antibody levels. IP-10 is a chemokine secreted in response to IFN-γ in order to attract monocytes, macrophages, T-cells and NK cells to infection sites (51) which are critical in containing an active CMV infection. Tie-2, also known as transmembrane receptor tyrosine kinase (Tek) is an anti-inflammatory angiogenesis factor which hinders vascular permeability hence leukocyte trafficking from vasculature to infected tissues (52). This may be a measure to control CMV-induced inflammation in a bid to regain immune homeostasis. CMV-exposed T-cells are known to produce IL-2 (53), which in turn promotes NK cell proliferation and cytotoxic function consequently controlling CMV viremia (54).

Our results show an increase of soluble factors such as VEGF and VEGF-C not only important for lymphanngiogenesis but are also important for tissue repair. In addition to elevation of cytokines such as IL4 and IL2 associated with immune regulation. Eotaxin, important in recruiting eosinophils to point of infection was elevated in cases. We speculate that the higher incidence of STIs in the cases may have been a major driver of the cytokine patterns observed. The skewing of immune responses to a Th2 profile probably is an attempt to immunoregulate STI related inflammation. This may however dampen Th1 responses that would have otherwise been important to protect against HIV-1 acquisition.

A major strength of this study is the use of a unique set of sample material that were carefully collected very close to the HIV-1 infection event, which enables us to clearly elucidate basal immune responses that are more likely to play a role in HIV-1 acquisition. However, our study is not without limitations. First, not all endemic pathogens could be measured, and an attempt to measure global pathogen exposure would still be limited to known pathogens. Second, cytokines may be secreted in localized immunological niches and in small quantities that are targeted for specific functions, which may have hindered our ability to measure these responses in plasma. Using advanced molecular -omics approaches, such as transcriptional analysis of the RNA encapsulated in small extracellular vesicles (sEVs) circulating in biological fluids such as plasma may be more representative of events occurring elsewhere in the body and reveal subtle but consequential differences between groups. Third, we assessed cytokine profiles approximately three months prior to HIV-1 acquisition. It is possible that the cytokine milieu closer to the date of infection may have been a better reflection of the extent of susceptibility to HIV-1 acquisition. However, taking into consideration the challenges of accurately estimating the date of infection, a three-month (+/-2) time point prior to HIV-1 acquisition is reasonable.

In conclusion, and consistent with literature, we observed that exposure to STIs was associated with increased risk for HIV-1 acquisition, which underscores the role of screening and STI treatment as crucial interventions towards controlling HIV-1 transmission among HIV-1 high risk populations. Importantly, our findings suggest that dampening of immune responses by an elevated Th2 associated profile may hinder optimum Th1 responses necessary for viral immunity, which may enhance susceptibility to HIV-1 acquisition. Immunomodulatory interventions aimed at inhibiting activation of Th2-associated pathways may have implications for HIV-1 prevention, over and above other STI control strategies and may reduce dampening of immune responses to vaccination.

## Data availability statement

The original contributions presented in the study are included in the article/**Supplementary Material**. Further inquiries can be directed to the corresponding author.

## Ethics statement

The studies involving humans were approved by Kenya Medical Research Institute - Scientific and Ethics Review Committee. The studies were conducted in accordance with the local legislation and institutional requirements. The participants provided their written informed consent to participate in this study.

## Author contributions

LF: Data curation, Formal analysis, Methodology, Writing – original draft, Writing – review & editing. CA: Supervision, Writing – review & editing. CS: Data curation, Methodology, Writing – review & editing. RB: Data curation, Methodology, Writing – review & editing. JH: Data curation, Methodology, Writing – review & editing. JE: Writing – review & editing. TN: Funding acquisition, Writing – review & editing. ES: Methodology, Resources, Writing – review & editing. AH: Data curation, Formal analysis, Methodology, Supervision, Writing – review & editing. EN: Conceptualization, Data curation, Funding acquisition, Investigation, Methodology, Resources, Supervision, Writing – review & editing.

## References

[1] National Institute of Allergy and Infectious Diseases. Global Research in Sub-Saharan Africa. (2023). Available at: https://www.niaid.nih.gov/research/niaid-research-sub-saharan-africa.

[2] Bonagura VR Rosenthal DW. Infections that cause secondary immune deficiency. Stiehm’s Immune Defic (2020), 1035–58. doi: 10.1016/B978-0-12-816768-7.00049-1

[3] Chen C Louie S Mccormick B Walker WA Shi HN. Concurrent infection with an intestinal helminth parasite impairs host resistance to enteric. Society (2005) 73(9):5468–81. doi: 10.1128/IAI.73.9.5468-5481.2005 PMC1231118 16113263

[4] van Riet E Hartgers FC Yazdanbakhsh M. Chronic helminth infections induce immunomodulation: Consequences and mechanisms. Immunobiology (2007) 212(6):475–90. doi: 10.1016/j.imbio.2007.03.009 17544832

[5] Moreau E Chauvin A. Immunity against helminths: Interactions with the host and the intercurrent infections. J BioMed Biotechnol (2010) 2010. doi: 10.1155/2010/428593 PMC2817558 20150967

[6] Maizels RM. Regulation of immunity and allergy by helminth parasites. Allergy (2020) 75(3):524–34. doi: 10.1111/all.13944 31187881

[7] Lemaitre M Watier L Briand V Garcia A Le Hesran JY Cot M. Coinfection with Plasmodium falciparum and Schistosoma haematobium: Additional evidence of the protective effect of schistosomiasis on malaria in Senegalese children. Am J Trop Med Hyg (2014) 90(2):329–34. doi: 10.4269/ajtmh.12-0431 PMC3919243 24323515

[8] Bradbury RS Piedrafita D Greenhill A Mahanty S. Will helminth co–infection modulate COVID–19 severity in endemic regions? Nat Rev Immunol (2020) 20(6):342–2. doi: 10.1038/s41577-020-0330-5 PMC7193760 32358579

[9] Kurup SP Obeng–Adjei N Anthony SM Traore B Doumbo OK Butler NS. Regulatory T cells impede acute and long–term immunity to blood–stage malaria through CTLA–4. Nat Med (2017) 23(10):1220–5. doi: 10.1038/nm.4395 PMC5649372 28892065

[10] Urban BC Cordery D Shafi MJ Bull PC Newbold CI Williams TN. The frequency of BDCA3–positive dendritic cells is increased in the peripheral circulation of Kenyan children with severe malaria. Infect Immun (2006) 74(12):6700–6. doi: 10.1128/IAI.00861-06 PMC1698077 17000725

[11] Bediako Y Adams R Reid AJ Valletta JJ Ndungu FM Sodenkamp J. Repeated clinical malaria episodes are associated with modification of the immune system in children. BMC Med (2019) 17(1):1–14. doi: 10.1186/s12916-019-1292-y 30862316 PMC6415347

[12] Illingworth J Butler NS Roetynck S Mwacharo J Pierce SK Bejon P. Chronic exposure to plasmodium falciparum is associated with phenotypic evidence of B and T cell exhaustion. J Immunol (2013) 190(3):1038–47. doi: 10.4049/jimmunol.1202438 PMC3549224 23264654

[13] Horne–Debets JM Faleiro R Karunarathne DS Liu XQ Lineburg KE Poh CM. PD–1 dependent exhaustion of CD8+ T cells drives chronic malaria. Cell Rep (2013) 5(5):1204–13. doi: 10.1016/j.celrep.2013.11.002 24316071

[14] Bates M Brantsaeter AB. Human cytomegalovirus (CMV) in Africa: a neglected but important pathogen. J Virus Erad . (2016) 2(3):136. doi: 10.1016/S2055-6640(20)30456-8 27482452 PMC4967964

[15] Reddehase MJ. Adverse immunological imprinting by cytomegalovirus sensitizing for allergic airway disease. Med Microbiol Immunol (2019) 208(3):469. doi: 10.1007/s00430-019-00610-z 31076879 PMC7086984

[16] Sanders EJ Thiong’o AN Okuku HS Mwambi J Priddy F Shafi J. High prevalence of Chlamydia trachomatis and Neisseria gonorrhoeae infections among HIV–1 negative men who have sex with men in coastal Kenya. Sex Transm Infect (2010) 86(6):440–1. doi: 10.1136/sti.2010.043224 20656722

[17] Caputo V Libera M Sisti S Giuliani B Diotti RA Criscuolo E. The initial interplay between HIV and mucosal innate immunity. Front Immunol (2023) 14:1104423. doi: 10.3389/fimmu.2023.1104423 36798134 PMC9927018

[18] Passmore JAS Jaspan HB Masson L. Genital inflammation, immune activation and risk of sexual HIV acquisition. Curr Opin HIV AIDS (2016) 11(2):156–62. doi: 10.1097/COH.0000000000000232 PMC6194860 26628324

[19] Masson L Passmore JAS Liebenberg LJ Werner L Baxter C Arnold KB. Genital inflammation and the risk of HIV acquisition in women. Clin Infect Dis (2015) 61(2):260–9. doi: 10.1093/cid/civ298 PMC4565995 25900168

[20] Galvin SR Cohen MS. The role of sexually transmitted diseases in HIV transmission . Vol. 2, Nature Reviews Microbiology. Nat Rev Microbiol (2004) p:33–42. doi: 10.1038/nrmicro794 15035007

[21] Michaud CM. Global burden of infectious diseases. Encycl Microbiol (2009) 1:444. doi: 10.1016/B978-012373944-5.00185-1

[22] Price MA Kilembe W Ruzagira E Karita E Inambao M Sanders EJ. Cohort profile: IAVI’s HIV epidemiology and early infection cohort studies in Africa to support vaccine discovery. Int J Epidemiol . (2020) 2020:1–10. doi: 10.1093/ije/dyaa100 PMC7938500 32879950

[23] Kamali A Price MA Lakhi S Karita E Inambao M Sanders EJ. Creating an African HIV clinical research and prevention trials network: HIV prevalence, incidence and transmission. PloS One (2015) 10(1). doi: 10.1371/journal.pone.0116100 PMC4300215 25602351

[24] Amornkul PN Karita E Kamali A Rida WN Sanders EJ Lakhi S. Disease progression by infecting HIV–1 subtype in a seroconverter cohort in sub–Saharan Africa. AIDS (2013) 27(17):2775–86. doi: 10.1097/QAD.0000000000000012 PMC3815107 24113395

[25] Sanders EJ Okuku HS Smith AD Mwangome M Wahome E Fegan G. High HIV–1 incidence, correlates of HIV–1 acquisition, and high viral loads following seroconversion among MSM. AIDS (2013) 27(3):437–46. doi: 10.1097/QAD.0b013e32835b0f81 PMC3929859 23079811

[26] Osier FHA Fegan G Polley SD Murungi L Verra F Tetteh KKA. Breadth and magnitude of antibody responses to multiple plasmodium falciparum merozoite antigens are associated with protection from clinical malaria. Infect Immun . (2008) 76(5):2240. doi: 10.1128/IAI.01585-07 18316390 PMC2346713

[27] EUROIMMUN Anti–IgG CMV ELISA Kit. Available at: https://www.euroimmun.com/products/productdetails/2570/2/145990/.

[28] V–PLEX Human Biomarker 40–Plex Kit. Available at: https://www.mesoscale.com/en/products/v–plex–human–biomarker–40–plex–kit–k15209d/.

[29] Hassan AS Hare J Gounder K Nazziwa J Karlson S Olsson L. A Stronger Innate Immune Response During Hyperacute HIV–1 Infection is associated with Acute retroviral syndrome. Clin Infect Dis (2021) 73(5):832–41. doi: 10.1093/cid/ciab139 PMC8423478 33588436

[30] Muema DM Akilimali NA Ndumnego OC Rasehlo SS Durgiah R Ojwach DBA. Association between the cytokine storm, immune cell dynamics, and viral replicative capacity in hyperacute HIV infection. BMC Med (2020) 18(1). doi: 10.1186/s12916-020-01529-6 PMC7093991 32209092

[31] Crowell TA Colby DJ Pinyakorn S Fletcher JLK Kroon E Schuetz A. Acute retroviral syndrome is associated with high viral burden, CD4 depletion, and immune activation in systemic and tissue compartments. Clin Infect Dis (2018) 66(10):1540–9. doi: 10.1093/cid/cix1063 PMC5930255 29228130

[32] Masha SC Wahome E Vaneechoutte M Cools P Crucitti T Sanders EJ. High prevalence of curable sexually transmitted infections among pregnant women in a rural county hospital in Kilifi, Kenya. PloS One (2017) 12(3). doi: 10.1371/journal.pone.0175166 PMC5375155 28362869

[33] Lillard JW Singh UP Boyaka PN Singh S Taub DD McGhee JR. MIP–1α and MIP–1β differentially mediate mucosal and systemic adaptive immunity. Blood (2003) 101(3):807–14. doi: 10.1182/blood-2002-07-2305 12393512

[34] Lee CG Link H Baluk P Homer RJ Chapoval S Bhandari V. Vascular endothelial growth factor (VEGF) induces remodeling and enhances TH2–mediated sensitization and inflammation in the lung. Nat Med . (2004) 10(10):1095–103. doi: 10.1038/nm1105 PMC3434232 15378055

[35] Chen L Grabowski KA Xin J Coleman J Huang Z Espiritu B. IL–4 induces differentiation and expansion of Th2 cytokine–producing eosinophils. J Immunol (2004) 172(4):2059–66. doi: 10.4049/jimmunol.172.4.2059 14764670

[36] Angelo LS Kurzrock R. Vascular endothelial growth factor and its relationship to inflammatory mediators. Clin Cancer Res (2007) 13(10):2825–30. doi: 10.1158/1078-0432.CCR-06-2416 17504979

[37] Reinders MEJ Sho M Izawa A Wang P Mukhopadhyay D Koss KE. Proinflammatory functions of vascular endothelial growth factor in alloimmunity. J Clin Invest (2003) 112(11):1655. doi: 10.1172/JCI17712 14660742 PMC281640

[38] Shukaliak JA Dorovini–Zis K. Expression of the β–chemokines RANTES and MIP–1β by human brain microvessel endothelial cells in primary culture. J Neuropathol Exp Neurol (2000) 59(5):339–52. doi: 10.1093/jnen/59.5.339 10888363

[39] Menten P Wuyts A Van Damme J. Macrophage inflammatory protein–1. Cytokine Growth Factor Rev . (2002) 13(6):455–81. doi: 10.1016/s1359-6101(02)00045-x 12401480

[40] Junttila IS. Tuning the cytokine responses: an update on interleukin (IL)–4 and IL–13 receptor complexes. Front Immunol (2018) 888:888. doi: 10.3389/fimmu.2018.00888 PMC6001902 29930549

[41] Koppensteiner H Brack–Werner R Schindler M. Macrophages and their relevance in Human Immunodeficiency Virus Type I infection. Retrovirology. BioMed Cent (2012) 9:1–11. doi: 10.1186/1742-4690-9-82 PMC3484033 23035819

[42] Levy JA. HIV and the pathogenesis of AIDS, third edition. Am Soc Microbiol (2007). doi: 10.1128/mr.57.1.183-289.1993

[43] Maksoud S El Hokayem J. The cytokine/chemokine response in Leishmania/HIV infection and co–infection. Heliyon (2023) 9(4):e15055. doi: 10.1016/j.heliyon.2023.e15055 37082641 PMC10112040

[44] McInally S Wall K Yu T Tirouvanziam R Kilembe W Gilmour J. Elevated levels of inflammatory plasma biomarkers are associated with risk of HIV infection. Retrovirology (2021) 18(1):1–11. doi: 10.1186/s12977-021-00552-6 33731158 PMC7968240

[45] Singh S Anshita D Ravichandiran V. MCP–1: Function, regulation, and involvement in disease. Int Immunopharmacol (2021), 107598. doi: 10.1016/j.intimp.2021.107598 34233864 PMC8135227

[46] Mascarau R Woottum M Fromont L Gence R Cantaloube–Ferrieu V Vahlas Z. Productive HIV–1 infection of tissue macrophages by fusion with infected CD4+ T cells. J Cell Biol (2023) 222(5):e202205103. doi: 10.1083/jcb.202205103 36988579 PMC10067447

[47] Snow RW Kibuchi E Karuri SW Sang G Gitonga CW Mwandawiro C. Changing malaria prevalence on the Kenyan coast since 1974: climate, drugs and vector control. PloS One (2015) 10(6). doi: 10.1371/journal.pone.0128792 PMC4479373 26107772

[48] Prakash D Fesel C Jain R Cazenave P-A Mishra GC Pied S. Clusters of cytokines determine malaria severity in plasmodium falciparum–infected patients from endemic areas of central India. J Infect Dis (2006) 2):198–207. doi: 10.1086/504720 16779726

[49] Waters LS Taverne J Tai PC Spry CJ Targett GA Playfair JH. Killing of Plasmodium falciparum by eosinophil secretory products. Infect Immun . (1987) 55(4):877. doi: 10.1128/iai.55.4.877-881.1987 3549562 PMC260432

[50] de Menezes MN Salles ÉM Vieira F Amaral EP Zuzarte–Luís V Cassado A. IL–1α promotes liver inflammation and necrosis during blood–stage Plasmodium chabaudi malaria. Sci Rep (2019) 9(1):1–12. doi: 10.1038/s41598-019-44125-2 31110285 PMC6527574

[51] Álvarez H Gutiérrez–Valencia A Mariño A Saborido–Alconchel A Calderón–Cruz B Pérez–González A. IP–10 and MIG are sensitive markers of early virological response to HIV–1 integrase inhibitors. Front Immunol (2023) 14:1257725. doi: 10.3389/fimmu.2023.1257725 37920466 PMC10619723

[52] Hughes DP Marron MB Brindle NPJ. The antiinflammatory endothelial tyrosine kinase Tie2 interacts with a novel nuclear factor–kappaB inhibitor ABIN–2. Circ Res (2003) 92(6):630–6. doi: 10.1161/01.RES.0000063422.38690.DC 12609966

[53] Becknell B Caligiuri MA. Interleukin–2, interleukin–15, and their roles in human natural killer cells. Adv Immunol (2005) 86:209–39. doi: 10.1016/S0065-2776(04)86006-1 15705423

[54] Maeker HT Maino VC. Analyzing T–cell responses to cytomegalovirus by cytokine flow cytometry. Hum Immunol (2004) 65(5):493–9. doi: 10.1016/j.humimm.2004.02.004 15172449

