# Supplementary material for: Exposure to common infections may shape basal immunity and potentially HIV-1 acquisition amongst a high-risk population in Coastal Kenya
Source: Front Immunol. 2024 Jan 11;14:1283559. doi: 10.3389/fimmu.2023.1283559 (PMC10808675; doi:10.3389/fimmu.2023.1283559)
Supplement: Supplementary file 1 [file DataSheet_1.pdf]

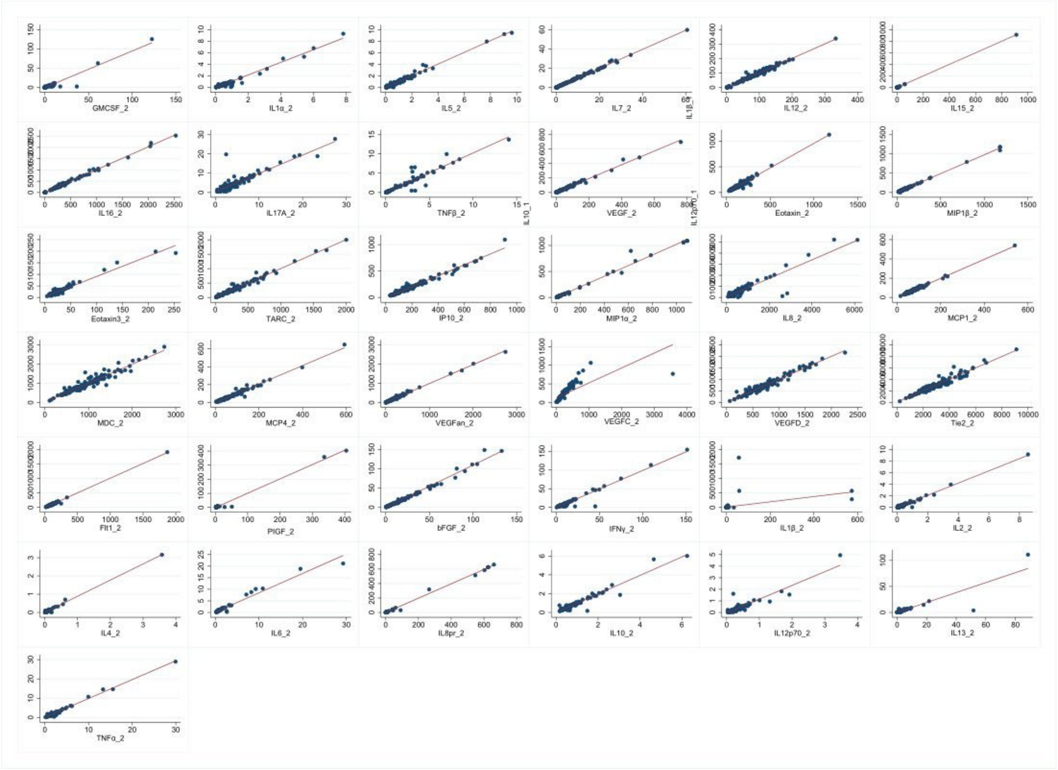

Supplementary Figure 1: Scatter plots illustrating correlation between the duplicate analyte read-outs from the MSD assay. Red line denotes the line of best fit. Each dot represents the concentration of the first replicate (y axis) and the second replicate (x axis) in each sample.

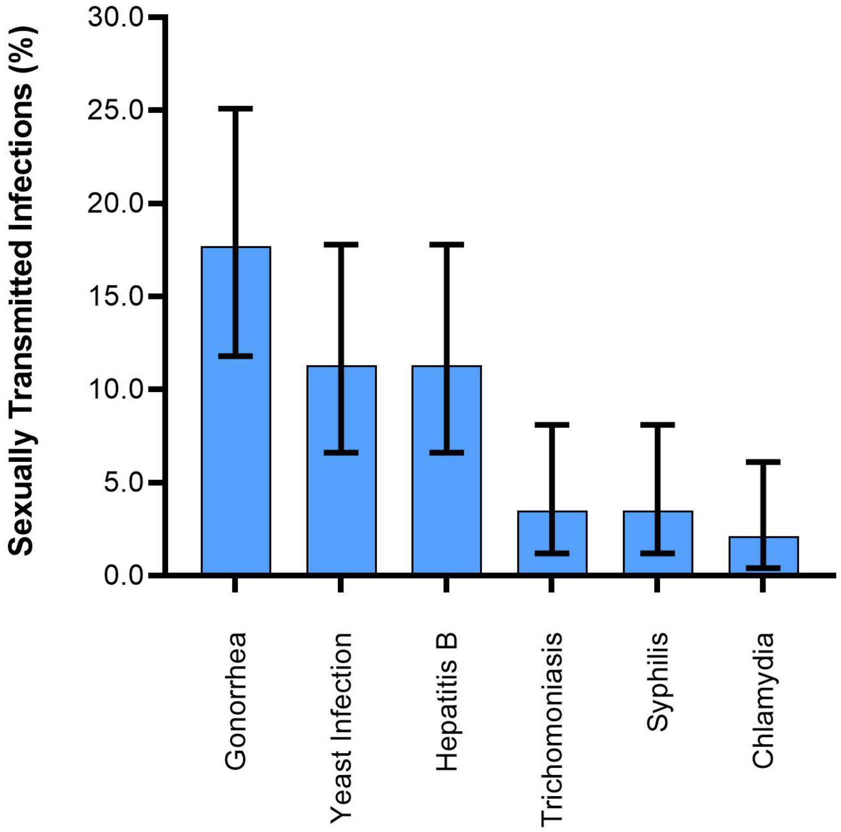

Supplementary Figure 2: Bar graph showing the prevalence of each of the six sexually transmitted infections (STIs) screened (n=141). Horizontal bars denote mean and 95% confidence interval.

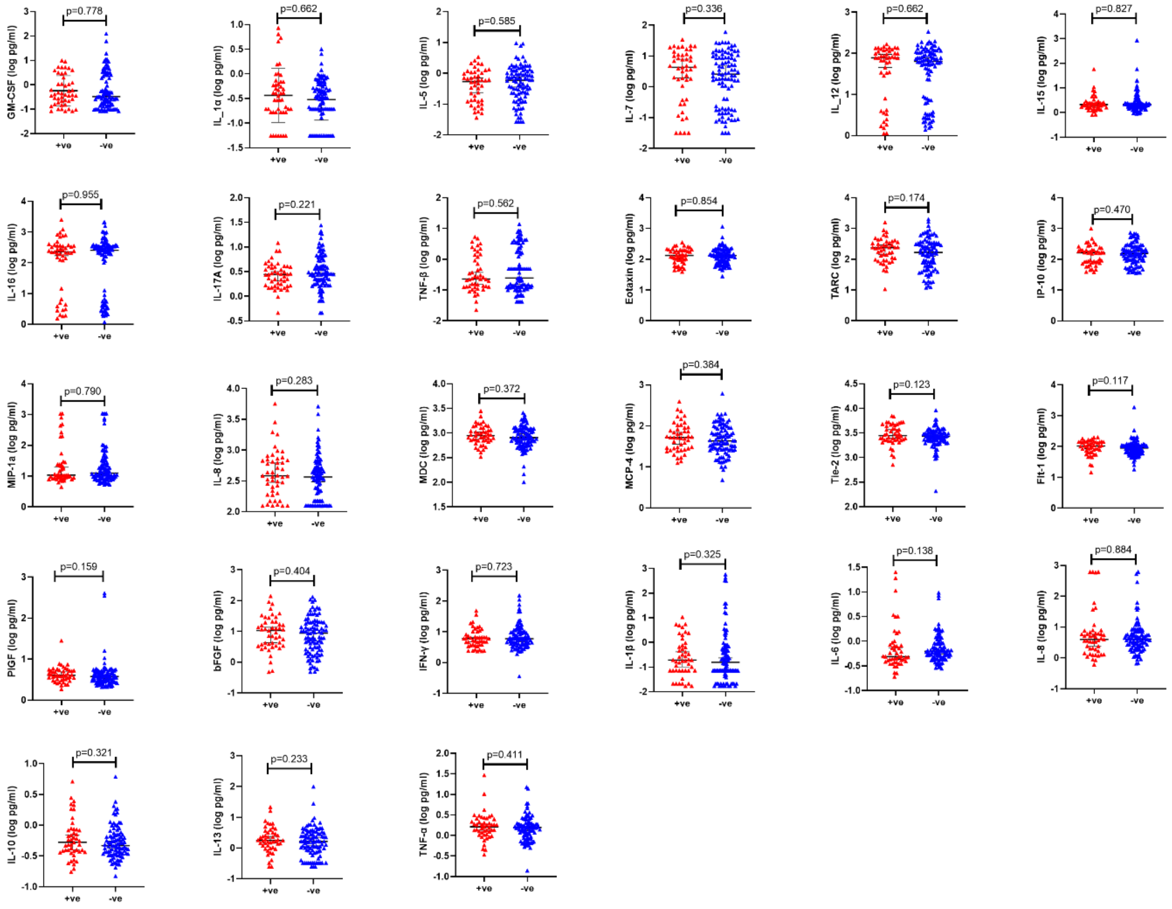

Supplementary Figure 3: Non-significantly different cytokine levels in HIV +ve (red): volunteers who contracted HIV-1 during follow up (cases) and HIV -ve (blue): volunteers who remained HIV-1 negative (controls). Comparison between HIV +ve and HIV -ve done by Wilcoxon rank-sum test, two-tailed, p<0.1 considered significant, median log pg/ml (95% confidence interval), n=141). [*VEGF (Vascular endothelial growth factor); MIP-1β (Macrophage Inflammatory Protein 1β); IL (Interleukin); MCP-1 (Monocyte Chemoattractant Protein 1)*]

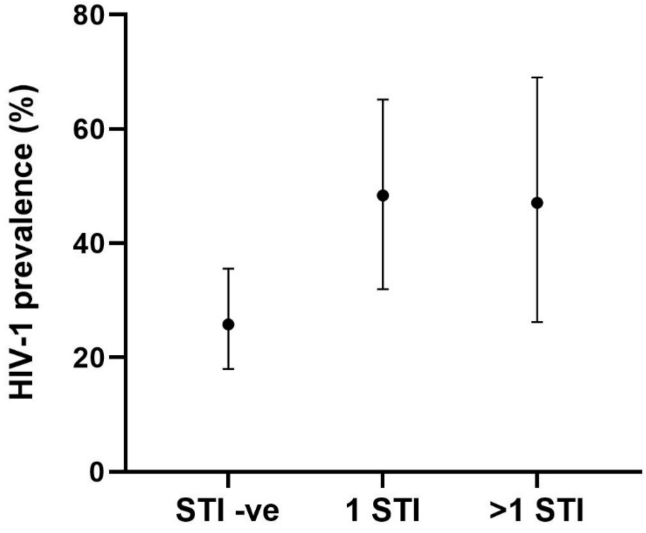

Supplementary Figure 4: Graph illustrating comparison of HIV-1 prevalence between volunteers with 0 vs 1 STI vs more than 1 STI, where STI -ve was defined as the lack of any STI, respectively in the last 6 months prior to HIV-1 incidence in cases. Horizontal bars denote mean and 95% confidence intervals.

SUPPLEMENTARY TABLES

Supplementary Table 1: Table showing correlation coefficients between duplicate observations for all 37 analytes. Values in bold represent analytes with r below 0.9 and the adjusted r values after removal of outliers (n=141).

Analyte	Correlation Coefficient (r)	P-value
GM-CSF	0.9625	<0.0001
IL-1α	0.9844	<0.0001
IL-5	0.9911	<0.0001
IL-7	0.9981	<0.00001
IL-12	0.9912	<0.0001
IL-15	0.9999	<0.001
IL-16	0.9974	<0.0001
IL-17A	0.9142	<0.0001
TNF-β	0.9555	<0.0001
VEGF	0.9949	<0.0001
Eotaxin	0.9804	<0.0001
MIP-1β	0.9984	<0.0001
Eotaxin-3	0.9764	<0.0001
TARC	0.9899	<0.0001
IP-10	0.9873	<0.0001
MIP-1α	0.9952	<0.0001
IL-8	0.9184	<0.0001
MCP-1	0.9930	<0.0001
MDC	0.9597	<0.0001
MCP-4	0.9882	<0.0001
VEGF	0.9971	<0.0001

VEGF-C	**0.7244**<br>**0.9794**	<0.0001
VEGF-D	0.9791	<0.0001
Tie-2	0.9644	<0.0001
Flt-1	0.9965	<0.001
PIGF	0.9943	<0.0001
bFGF	0.9929	<0.0001
IFN-γ	0.9745	<0.0001
IL-1β	**0.3825**<br>**0.9306**	<0.0001<br><0.0001
IL-2	0.9905	<0.0001
IL-4	0.9945	<0.0001
IL-6	0.9807	<0.0001
IL-8	0.9981	<0.0001
IL-10	0.9672	<0.0001
IL-12p70	0.9294	<0.0001
IL-13	**0.8800**<br>**0.9510**	<0.0001<br><0.0001
TNF-α	0.9928	<0.0001

Supplementary Table 2: Table showing correlation coefficients between malaria/CMV-specific antibodies and analyte concentrations (n=117).

Analyte	Correlation with Malaria antibodies		Correlation with CMV antibodies	
	Correlation Coefficient (r)	P-value	Correlation Coefficient (r)	P-value
GM-CSF	-0.13	0.163	0.04	0.668
IL-1α	0.11	0.260	-0.03	0.722
IL-5	0.09	0.337	-0.07	0.439
IL-7	0.14	0.138	-0.03	0.785
IL-12	0.15	0.119	0.10	0.261
IL-15	-0.13	0.166	0.007	0.939
IL-16	0.07	0.470	0.02	0.846
IL-17A	0.01	0.911	-0.01	0.890
TNF-β	-0.15	0.096	-0.01	0.889
VEGF	0.12	0.189	-0.03	0.740
Eotaxin	-0.11	0.242	-0.03	0.754
MIP-1β	0.04	0.698	0.01	0.897
Eotaxin-3	-0.04	0.654	-0.07	0.439
TARC	0.16	0.093	-0.09	0.349

IP-10	-0.09	0.309	0.03	0.749
MIP-1α	0.18	0.053	-0.05	0.626
IL-8	0.11	0.220	-0.01	0.903
MCP-1	0.01	0.874	-0.07	0.468
MDC	0.11	0.230	0.02	0.850
MCP-4	-0.05	0.590	-0.04	0.674
VEGF	0.02	0.855	-0.06	0.540
VEGF-C	-0.07	0.453	-0.04	0.656
VEGF-D	-0.06	0.546	0.02	0.824
Tie-2	-0.08	0.388	-0.09	0.338
Flt-1	-0.002	0.981	-0.05	0.573
PIGF	-0.0002	0.999	-0.07	0.477
bFGF	-0.01	0.925	-0.04	0.693
IFN-γ	-0.05	0.600	0.04	0.692
IL-1β	0.05	0.628	-0.08	0.370
IL-2	0.03	0.785	-0.07	0.475
IL-4	0.18	0.054	-0.04	0.707
IL-6	0.01	0.874	-0.03	0.721
IL-8	-0.02	0.800	-0.003	0.977
IL-10	0.24	0.059	-0.12	0.201

IL-12p70	0.08	0.374	-0.08	0.390
IL-13	-0.10	0.276	0.01	0.915
TNF-α	0.04	0.674	-0.02	0.833

Supplementary Table 3: Distribution of log_{10} median analyte concentrations showing differences between malaria seropositive and seronegative volunteers, and CMV titres above the median (CMV high) versus below the median (CMV low) levels (n=117).

**Analyte**	**Malaria seropositive (log_{10} median pg/ml)**	**Malaria seronegative (log_{10} median pg/ml)**	**P-value**	**CMV high (log_{10} median pg/ml)**	**CMV low (log_{10} median pg/ml)**	**P-value**
GM-CSF	-0.40	0.29	0.089	-0.31	-0.39	0.446
IL-1α	-0.39	-0.58	0.034	-0.44	-0.40	0.674
IL-5	-0.11	-0.62	0.023	-0.27	-0.21	0.491
IL-7	0.68	-0.06	0.093	0.67	0.55	0.792
IL-12	1.89	1.36	0.086	1.82	1.84	0.942
IL-15	0.29	0.34	0.091	0.30	0.32	0.698
IL-16	2.35	1.586	0.092	2.35	2.33	0.864
IL-17A	0.45	0.41	0.287	0.44	0.45	0.737
TNF-β	-0.82	-0.05	0.081	-0.81	-0.71	0.787
VEGF	1.60	0.88	0.072	1.51	1.34	0.851
Eotaxin	2.07	2.11	0.377	2.08	2.12	0.653
MIP-1β	1.89	1.85	0.912	1.90	1.85	0.316
Eotaxin-3	1.37	1.30	0.183	1.33	1.34	0.806
TARC	2.38	2.31	0.872	2.31	2.39	0.321

IP-10	2.11	2.19	0.217	2.11	2.20	0.017
MIP-1α	1.06	1.07	0.918	1.05	1.08	0.963
IL-8	2.61	2.61	0.652	2.64	2.57	0.298
MCP-1	1.80	1.79	0.960	1.77	1.82	0.110
MDC	2.97	2.90	0.941	2.96	2.94	0.380
MCP-4	1.65	1.74	0.327	1.72	1.72	0.759
VEGF	2.10	2.07	0.946	2.11	2.09	0.950
VEGF-C	2.24	2.36	0.311	2.28	2.27	0.554
VEGF-D	2.77	2.87	0.204	2.87	2.77	0.073
Tie-2	3.41	3.44	0.399	3.39	3.45	0.017
Flt-1	1.99	1.99	0.731	1.94	2.00	0.110
PIGF	0.57	0.63	0.105	0.60	0.58	0.472
bFGF	1.02	1.12	0.511	1.01	1.07	0.173
IFN-γ	0.79	0.79	0.701	0.79	0.81	0.562
IL-1β	-1.01	-0.93	0.838	-1.04	-0.99	0.679
IL-2	-0.72	-0.81	0.514	-0.75	-0.72	0.034
IL-4	-1.34	-1.34	0.270	-1.37	-1.31	0.474
IL-6	-0.21	-0.22	0.543	-0.20	-0.22	0.784
IL-8	0.62	0.60	0.853	0.66	0.59	0.581
IL-10	-0.26	-0.33	0.024	-0.36	-0.23	0.403
IL-12p70	-0.71	-0.85	0.057	-0.74	-0.71	0.522
IL-13	0.26	0.23	0.849	0.25	0.27	0.805
TNF-α	0.18	0.10	0.115	0.15	0.18	0.663

Supplementary Table 4: Table showing number and percentage of volunteers who tested positive for one and more than one STI (n=141).

**Sexually transmitted infection**	**Frequency (n/N)**	**Percent (%)**
Gonorrhea	25	17.7
Hepatitis B	16	11.3
Yeast Infection	16	11.3
Trichomoniasis	5	3.5
Syphilis	5	3.5
Chlamydia	3	2.1
Gonorrhea + Yeast Infection	4	2.8
Gonorrhea + Hepatitis B	3	2.1
Gonorrhea + Syphilis	1	0.7
Gonorrhea + Chlamydia	1	0.7
Yeast Infection + Syphilis	1	0.7
Gonorrhea + Yeast Infection + Trichomoniasis	2	1.4
Gonorrhea + Hepatitis B + Yeast Infection	1	0.7
Gonorrhea + Yeast Infection + Syphilis	1	0.7
Gonorrhea + Hepatitis B + Yeast Infection	1	0.7
Yeast Infection + Syphilis + Hepatitis B	1	0.7

Supplementary Table 5: Table showing number of volunteers who tested positive per infection and those who tested positive for more than one kind of infection.

**Infections**	**Frequency (n/N)**	**Percent (%)***
**Malaria**	81	69.2
**CMV**	115	98.3
**Any STI****	48	34.0
**Malaria + CMV**	79	67.5
**Malaria + Any STI**	21	17.9
**CMV + Any STI**	35	29.9
**Malaria + CMV + Any STI**	21	17.9

*Percentages based on N=117 which is the number of samples which were available for measurements of exposure to malaria and CMV.

**Any STI analysis was based on the full dataset of N=141 as STI data was extracted from the historic cohort.
